# Ultrasound-Assisted Extraction of Isoquercetin from *Ephedra alata* (Decne): Optimization Using Response Surface Methodology and In Vitro Bioactivities

**DOI:** 10.3390/antiox12030725

**Published:** 2023-03-15

**Authors:** Ezzouhra El Maaiden, Nagib Qarah, Amine Ezzariai, Adil Mazar, Boubker Nasser, Khadija Moustaid, Hassan Boukcim, Abdelaziz Hirich, Lamfeddal Kouisni, Youssef El Kharrassi

**Affiliations:** 1 African Sustainable Agriculture Research Institute (ASARI), Mohammed VI Polytechnic University (UM6P), Laayoune 70000, Morocco; amine.ezzariai@um6p.ma (A.E.); adil.mazar@um6p.ma (A.M.); hassan.boukcim@um6p.ma (H.B.); abdelaziz.hirich@um6p.ma (A.H.); lamfeddal.kouisni@um6p.ma (L.K.); 2 Department of Chemistry, Faculty of Education-Zabid, Hodeidah University, Hodeidah P.O. Box 3114, Yemen; nagib2015qarah@gmail.com; 3 Laboratory of Biochemistry, Neurosciences, Natural Resources and Environment, Hassan I University of Settat, BP 577, Settat 26000, Morocco; boubker.nasser@uhp.ac.ma; 4 Laboratory of Applied Chemistry and Environment, Hassan I University of Settat, BP 577, Settat 26000, Morocco; khadija.moustaid@uhp.ac.ma

**Keywords:** *Ephedra alata*, isoquercetin, response surface methodology, skin aging, ultrasound extraction

## Abstract

Isoquercetin (ISQ) is reported to be a powerful antioxidant with extremely high bioavailability and structural stability compared to aglycone quercetin. Despite this, it is not well studied due to the limited methods for its extraction. With the growing interest in the research and analysis of ISQ-rich herbs, there is a need to optimize an efficient and rapid method for their extraction. In the present study, the ultrasound-assisted extraction of ISQ from *Ephedra alata* Decne was optimized by a response surface methodology (RSM) using high-performance liquid chromatography as a separation method. The best possible ranges for extraction time (10–30 min), temperature (50–70 °C), ultrasonic power (60–90 W), solvent-to-solid ratio (50–70 mL/g), and ethanol concentration (50–70%) were determined using a single factor analysis. Subsequently, an optimization of the extraction conditions was performed with RSM using the Box–Behnken design. An ultrasonication time of 10 min, a temperature of 60 °C, a power of 75 W, a solvent-to-solid ratio of 60 mL/g, and an ethanol concentration of 70% were determined to be the optimal conditions for the highest recovery of isoquercetin (1033.96 ± 3.28 µg/g). Furthermore, *E. alata* powder morphology (using a scanning electron microscope), antioxidant activities, and the inhibition potential of key enzymes involved in skin aging (elastase and collagenase), hyperpigmentation (tyrosinase), diabetes (α-amylase), inflammation (hyaluronidase), and neurodegenerative disorders (cholinesterase) were determined and compared with those using the Soxhlet method. This study established a highly efficient method for ISQ extraction and suggested several potential applications of ISQ in the pharmaceutical and cosmetics industries.

## 1. Introduction

**Figure 1:**
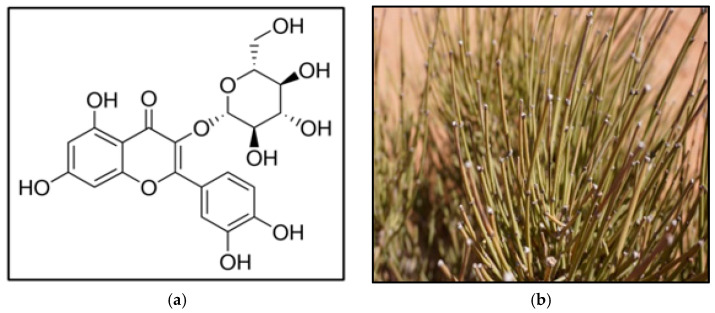
(**a**) Structure of quercetin 3-glucoside (isoquercetin, ISQ)*,* and morphology of *E. alata* (**b**).

Isoquercetin (ISQ) (quercetin-3-O-glucoside, Figure 1a) is one of the main glycosidic groups of quercetin, a naturally occurring flavonol [1]. Biological research on quercetin has been extensively documented over the past decades [2]. Recently, ISQ has gained considerable attention as a prospective compound because it has superior water solubility (95 mg/L) compared with quercetin [3]. However, the biological activity of quercetin glycosides is not well studied. The Web of Science database listed 36,072 references on quercetin, with 11,428 references on rutin and only 369 references on ISQ [4]. Regardless, ISQ has increasingly attracted the attention of researchers because of its higher bioavailability compared to quercetin. In addition, ISQ exhibits a variety of chemoprotective effects in vitro and in vivo on oxidative stress, cancer, cardiovascular disease, diabetes, and chronic allergic diseases [5]. Following the oral application of ISQ, the compound is metabolized to a great extent in the intestine and liver, with only minor amounts of undamaged ISQ being recovered in both plasma and tissues. The growing interest in researching and analyzing ISQ-rich herbs requires the optimization of an efficient and rapid extraction method.

The extraction of bioactive compounds from the raw materials of plants is a crucial step. Currently, several advanced extraction methods are being developed, including the ultrasound-assisted extraction (UAE) method, in which ultrasonic waves are used to separate components from the raw material or herbs [6]. The cavitation force generated by the ultrasonic waves leads to the disintegration of cell walls and the accelerated release of matrix components [7]. In addition, several other parameters affect the efficiency of these extraction methods, such as solvent concentration, material-to-solvent ratio, temperature, duration, and ultrasonic extraction power [8]. Therefore, the optimization of these parameters should be performed to improve the extraction efficiency of ISQs and preserve their bioactivities. The response surface method (RSM) is a statistical analytical method that has been widely applied to optimize the extraction of bio-compounds from various vegetable materials using UAE techniques [8,9].

*Ephedra alata* Decne (family *Ephedraceae*), with its strong pine fragrance and astringent flavor, is mainly distributed in arid and semiarid regions around the world [10]. Traditionally, it is used to treat kidney disorders, bronchial asthma, and circulatory and digestive system disorders as well as to manage cancer [10]. Previous studies have documented that this plant has several biological activities, such as antibacterial activity and protection against cancer, liver, and cardiovascular diseases [11]. The medicinal properties of the plant under study are attributed to secondary metabolites, which include glycosides, flavonoids, phenolic compounds, and alkaloids. ISQ is a predominant component of quercetin in *E. alata*. This study evaluated the influence of various extracting parameters on the recovery of ISQ from *E. alata,* with the objective of optimizing ultrasonic extraction techniques. Several extraction conditions were evaluated to identify the conditions most conducive to achieving the highest yield of ISQ and improving product bioactivities.

## 2. Materials and Methods

### 2.1. Plant Material

The aerial parts of *Ephedra alata* Decne were gathered in July 2021 from Laâyoune Sakia El Hamra region, Morocco (Figure 1b), using standard fieldwork and collection practices [12,13]. The plant was authenticated and identified by taxonomists and deposited at the local ASARI herbarium, Um6p (Morocco). Plant material was dried at 60 °C in a tray dryer for 72 h. Dried materials were ground to 250–500 μm and then stored in an airtight container (4 °C) until further utilization.

### 2.2. Ultrasound-Assisted Extraction

Ultrasound-assisted extraction (UAE) of *E. alata* was carried out with a sonicator (QSonica Q500, 500 W power, 20 kHz, 25-mm probe, 120 μm maximum amplitude) under controlled time, temperature, amplitude, and pulse. The *E. alata* powder was mixed well with the ethanol/formic acid solution according to the liquid-to-solid (L/S) ratio. Then, the mixture was sonicated at a specified amplitude for a specified extraction time. It was subsequently pelleted at 2600× *g* for 15 min, strained with a paper filter (Whatman No. 1, England, UK), and concentrated in a vacuum rotary evaporator.

### 2.3. Soxhlet Extraction

The extraction process was performed using a Soxhlet apparatus with 3 g of sample powder and 120 mL of ethanol: water solution (70:30 *v*:*v*) for a period of 6 h. Following this, the solvent was evaporated using a rotary evaporation apparatus equipped with a thermostat, prior to being filtered off with a 0.45 μm filter for chromatographic analysis.

### 2.4. Single-Factor Experimental Design

A single-factor experimental design (SFED) was adopted to identify and select appropriate values from the 5 variables tested: the ethanol concentration (20, 30, 40, 50, 60, 70%), the solvent–solid report (20, 30, 40, 50, 60, 70 mL/g), the ultrasonic power (30, 60, 75, 90, 115, 130, 150, 180 W), the duration (5, 10, 15, 20, 25, 30, 35, 40, 60, 80, 100, 120 min), and the temperature (30, 40, 50, 60, 70, 80 °C). The common conditions selected were as follows: an ethanol concentration of 70%; a liquid-to-material ratio of 30 mL/g; an ultrasonic power of 90 W; an ultrasonic time of 10 min; and an ultrasonic temperature of 50 °C. One independent parameter was modified, and the others were held constant. ISQ was quantified with high-performance liquid chromatography (HPLC) to select the relevant independent parameters that significantly influenced the efficiency of the extraction.

### 2.5. Experimental Design and Statistical Analysis

Following the results of the SFED experiments, the optimization conditions of sample (*E. alata* ISQ) extraction were conducted by response surface methodology (RSM) using a Box–Behnken Design (BBD). A summary of the effective extraction parameters, including ultrasonic time (min, X1), ultrasonic temperature (°C, X2), solvent-to-solid ratio (mL/g, X3), ultrasonic power (W, X4), and the EtOH concentration (%, X5) are reported in Table 1 at 3 levels (−1, 0, 1). A total of 46 experimental cycles were performed (46 runs, *n* = 3). The response variable was the yield of ISQ (Y), as quantified by HPLC analysis. The design of the experimental runs and the results of the optimization of the ISQ extraction conditions are presented in Table 1. According to the ANOVA analysis, a *p*-value < 0.05 signifies that the tested conditions were significant, while a *p*-value < 0.0001 signifies that they were highly significant. Furthermore, a *p*-value > 0.10 signifies that the conditions were not significant. The change in the response value (Y) based on the five parameters was adjusted by an RSM, as illustrated by the equation below:

$$y=\beta 0+\sum_{i=1}^{k}\beta i\;xi+\sum_{i=1}^{k}\beta ii\;xi2\;+\sum_{i}^{k-1}\sum_{j}^{k}\beta ij\;xi\;xj$$

where *xi* and *xj* represent the independent parameters affecting the dependent response Y (ISQ), and *β*0, *βi*, *βii*, *βij*, and *k* represent the regression coefficients for the intercept, linear, quadratic, interaction terms, and the number of variables, respectively.

### 2.6. High-Performance Liquid Chromatography (HPLC) Analysis

The samples were analyzed using an Agilent 1200 HPLC system (Agilent Technologies, Santa Clara, CA, USA). Sample separation was performed on a Capcell PAK C18 column (internal diameter 250 × 4.6 mm, particle size 5 um) at 40 °C. A flow rate of 1 mL/min was used, and the injection volume was 10 µL. The mobile phase consisted of (A) water: formic acid (95:5, *v*/*v*) and (B) 100% methanol. The binary gradients were as follows: 0–25 min, 20–60% B; 25–25.1 min, 60–100% B; 25.1–30 min, 100–60% B; 30–30.1 min, 60–20% B; and 30.1–35 min, 20% B. The absorbance was measured at 360 nm. Identification of ISQ was conducted based on spectrum, standards, and previous studies. The results are presented in micrograms per gram dry weight (µg/g DW) [14]. Liquid chromatography-mass spectrometry (LC-MS) (Agilent Technologies, LC/MSD VL Series 1100 system, Palo Alto, CA, USA) analysis was conducted using an identical HPLC column and mobile phase. The Electrospray ionization mass spectrometer was run under negative ion mode using a drying gas flow rate (N2), drying gas temperature, nebulization pressure, and capillary voltage of the fragmentation of 10 L/min, 350 °C, 40 psi, 3.5 kV, and 110 V, respectively. The ions underwent scanning mode detection, and the mass range was set from 400 to 1400.

### 2.7. Antioxidant Assays

Three complementary assays were used to assess antioxidant potential (ABTS, FRAP, and DPPH) and were reported as Trolox equivalents per gram of sample (mg TE/g). Values were reported as average ± SD and have been shown to be significant at *p* < 0.05. Detailed protocols have been described for all assays in our previous work [15].

### 2.8. Enzyme Inhibition Assays

The pharmaceutical and cosmetic properties of ISQ as well as its potential to inhibit key enzymes involved in hyperpigmentation, skin aging, diabetes, inflammation, and neurodegenerative diseases (elastase and collagenase, tyrosinase, α-amylase, hyaluronidase, cholinesterase, and A*β*_1–42_ aggregations respectively) were evaluated and compared with ISQ extracted with the Soxhlet method. Detailed protocols have been described for all assays in our previous work [15].

#### 2.8.1. Tyrosinase Inhibition Assay

Anti-tyrosinase activity was evaluated using L-3,4dihydroxyphenylalanine as a substrate based on previous work [16]. The reaction was started by adding 40 µL of L-3,4dihydroxyphenylalanine (2 mM), and the inhibition of tyrosinase was evaluated by tracking the absorbance at 492 nm. Detailed protocols have been described for all assays in our previous work [15].

#### 2.8.2. Collagenase Inhibition Assay

Anti-collagenase activity was determined according to the method described by Wang et al. [17] with some modifications. One milligram of azo dye-impregnated collagen was weighed into tubes and then mixed with 800 µL of Tris-HCl (0.1 M, pH 7) plus 100 µL of the sample. Next, 100 µL of collagenase (200 units/mL) was added immediately to the resulting mixture and incubated at 43 °C for 1 h. Then, the samples were subjected to centrifugation at 3000 rpm for a period of 10 min. The supernatant liquid from each tube was placed on a 96-well plate, and the absorbance of all sample tubes was measured at 550 nm.

#### 2.8.3. Elastase Inhibition Assay

Anti-elastase activity has been detected by tracking p-nitroanilide release during N-succinyl-Ala-Ala-p-nitroanilide cleavage [18]. The reaction mixture was initiated with the addition of the substrate, and the anti-elastase activity was determined at 410 nm. Detailed protocols have been described for all assays in our previous work [15].

#### 2.8.4. Hyaluronidase Inhibition Assay

The anti-hyaluronidase activity was determined using the method as reported by Abhijit and Manjushree [19], in which 10 µL of bovine testicular hyaluronidase type 1-S (4200 units/mL) was dissolved in acetate buffer (0.1 M, pH 3.5) with 50 µL of samples, placed in a water bath at 37 °C, and incubated for 20 min. Hyaluronidase-activated Ca^2+^ was treated with 50 µL of sodium hyaluronate (12 mg/mL) dissolved in acetate buffer (0.1 M, pH 3.5) and incubated in a water bath (37 °C, 40 min). Next, 10 µL of sodium hydroxide (0.9 M) and 20 µL of sodium borate (0.2 M) were added to the mixture and incubated for 3 min in a boiling water bath. Next, 50 µL of ρ-dimethyl amino benzaldehyde solution (0.25 g of p-dimethyl amino benzaldehyde) dissolved in 21.88 mL of acetic acid (100%) and 3.12 mL of hydrochloric acid (10 N) was added. Tannic acid was used as the reference standard, and the absorbance was measured at 585 nm.

#### 2.8.5. α-Amylase Inhibition Assay

To evaluate the potential for α-amylase inhibition on 96-well plates, 25 µL of the sample was mixed with 50 µL of starch solution (0.05%) and incubated (30 °C, 10 min). Then, the reaction was completed with the addition of a combination of hydrogen chloride (25 µL, 1 M) and potassium iodide solution (100 µL). Absorbance measurements were performed at 630 nm, and the obtained data were expressed in acarbose equivalent (mmol ACE/g) [20].

#### 2.8.6. Cholinesterase Inhibition Assays

The in vitro anticholinesterase activities (acetylcholinesterase-AchE and butyrylcholinesterase- BchE) were assayed as previously reported [15]. For AchE and BchE, respectively, each reaction was initiated by adding 25 µL of acetyl thiocholine iodide (1.5 mM) or butyryl thiocholine chloride (4 mM) and then incubated at 25 °C for 10 min. Absorbance was measured at 405 nm. The AchE and BchE inhibition capacities are reported as milligrams of galantamine equivalents (mg GALAE/g).

#### 2.8.7. Aβ_1–42_ Aggregation Inhibition Assay

The Aβ_1–42_ aggregation inhibition assay was evaluated based on the thioflavin T florescence method, as described by Li et al. [21]. Briefly, freshly prepared solutions of Aβ_1–42_ (1 mM, dissolved in DMSO) were incubated with phosphate-buffered saline (PBS) (10 mM, pH 7.4) containing 10 mM NaCl in an ultrasonic bath for 1 min. The peptide solution (final Aβ concentration of 50 μM) was incubated alone or with 100 μg/mL of each sample at 37 °C for 48 h. Next, 80 μL of the test solution was diluted to 600 μL with PBS (containing 10 μM thioflavin T). Absorbance measurement was performed at 485 nm, and the result of Aβ_1–2_ aggregation inhibition was obtained using the following formula:

$$A\beta_{1–42}\;aggregation\;inhibition\;\left(\%\right)=\left[\left(1-F_{1}\right)/F_{0}\right]*100$$

where *F*_0_ and *F*_1_ are the florescence of the Aβ_1–42_ at 485 nm in the presence and absence of the inhibitors, respectively.

### 2.9. Scanning Electron Micrographs

The morphology of *E. alata* powder was examined with a scanning electron microscope (ETEC Corp., Hayward, CA, USA) after different treatments: powder before and after extraction with Soxhlet (SE), or our UAE-optimized method (UAE-optimized). After being fixed to a metal grid with double-sided adhesive tape, the powder was coated with gold under vacuum (20 µm, 400× magnification) and then analyzed with a scanning electron microscope at 2500× magnification.

### 2.10. Statistical Analysis

A one-way analysis of variance (ANOVA) and Tukey’s test was performed for the statistical evaluations of the data generated from the single-factor experimental design (SFED). Response surface methodology (RSM) optimization and modeling were performed in Design Expert Version 7.0.0 (Stat-Ease, Inc., Minneapolis, MN, USA). Analyses were performed in triplicate (*n* = 3).

## 3. Results and Discussion

### 3.1. Single-Factor Analysis of ISQ Extraction

It has been reported that different extraction parameters influence the extraction efficiency of quercetin glucoside derivatives from various plant matrices [22]. Here, using a single-factor experimental design (SFED), we evaluated the effect of five independent parameters (EtOH concentration, liquid–solid ratio, extraction time, extraction temperature, and ultrasonic power) on the extraction efficiency of ISQ from *E. alata*. The objective of this preliminary experiment was to identify the limiting extraction parameters.

#### 3.1.1. Effect of Ethanol Concentration on ISQ Extraction

**Figure 2:**
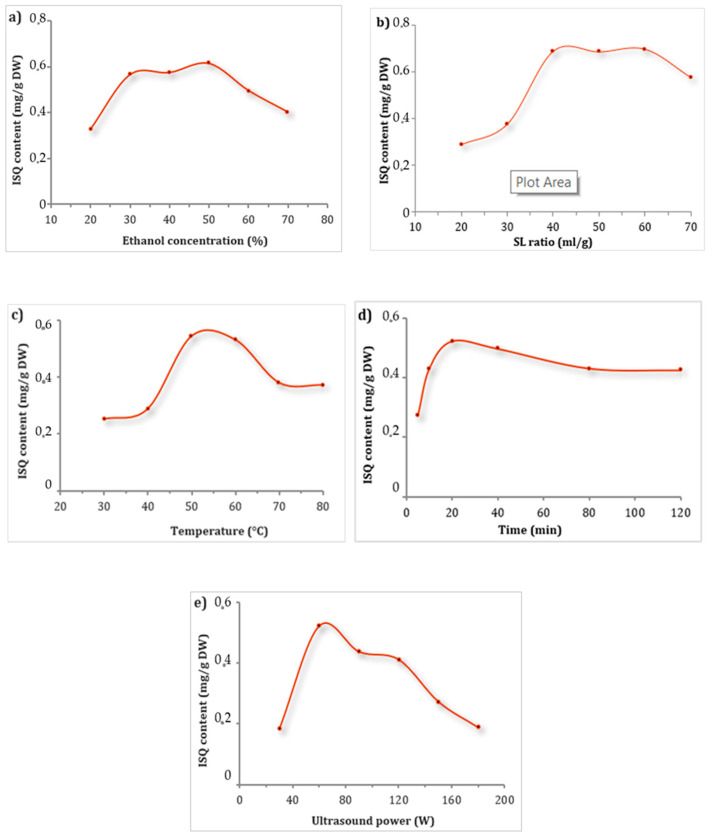
The effects of UAE parameters on the recovery of ISQ (mg/g) from *E. alata* in single-factor experiments: ethanol concentration (**a**), solid–liquid ratio (**b**), ultrasonic temperature (**c**), ultrasonic time (**d**), ultrasound temperature (**e**), and ultrasound power.

An important parameter to consider when designing an extraction procedure is the solvent used. In ultrasound-assisted extraction (UAE), the solvents used for the extraction of bioactive components from the plants are typically a combination of organic and aqueous solutions with different ratios [23]. Many mixtures, including acids (acetic, formic, and hydrochloric), ethanol, methanol, and water, are widely used to extract quercetin from plants [24]. In this study, given that our goal was to apply these ISQs for future cosmetic and pharmaceutical uses and to develop a green chemistry extraction method, EtOH was chosen as a solvent. EtOH is a less toxic solvent for humans and is more environmentally friendly than other organic solvents (e.g., methanol) [25]. Furthermore, its extractability can be adjusted by adding formic acid (CH_2_O_2_), making it an ideal solution for ISQ extraction. Interestingly, these two universal solvents (i.e., ethanol and formic acid) have been widely used in various cosmetic and/or food applications [26]. In this work, the extractability of ethanol and formic acid in mixtures with different proportions (20, 30, 40, 50, 60, and 70% ethanol) was evaluated. It was found that the ethanol content had a marked effect on the extraction of ISQ from *E. alata* (Figure 2a). The quantity of ISQ initially increased with increasing ethanol content, followed by a decrease with increasing ethanol ratio. This may be related to the polarity of ISQ [26]. As a result, the quantity of ISQ in the following experiments was maximized within the 30–50% ethanol ratios.

#### 3.1.2. Effect of Solid–Liquid Ratio on ISQ Extraction

The extraction efficiency of ISQ was significantly influenced by the solvent-to-solid ratio. As shown in Figure 2b, by increasing the solvent–solid ratio, the amount of extracted ISQ increased. The maximum amount of ISQ was obtained with the 40 mL/g ratio. However, when the solvent–solid ratio was higher than 60 mL/g, the amount of ISQ extracted decreased. This may be related to the decrease in the amount of powder to be wetted and the reduced contact area between the liquid and the material when the solvent ratio increases [27]. In this case, the maximum ISQ had been extracted after the liquid-to-material ratio had reached a certain level, and a further increase in the amount of solvent would result in waste. Therefore, the following experiments were maximized within the 40–60 mL/g solid/liquid ratio.

#### 3.1.3. Effect of Ultrasonic Temperature on ISQ Extraction

In this study, the influence of several extraction temperatures (30, 40, 50, 60, 70, and 80 °C) on the extraction of ISQ was evaluated (Figure 2c). The amount of extracted ISQ increased with increasing temperature. When the temperature was 50 °C, the extracted amount of ISQ reached its maximum. Thereafter, the extraction rate of ISQ decreased with increasing temperature. This may be mainly because increasing the temperature to a certain level improves the release of ISQ. A higher temperature decreases the surface tension and viscosity of the solvent–sample mixtures, which improves the extraction yield [28,29]. However, increasing the temperature to an excessive level led to the destruction or volatilization of ISQ, especially if the extraction time was longer [28,29,30]. Havlikova and Mikova revealed that temperatures above 70 °C cause rapid degeneration of the polyphenols; therefore, it is essential to carefully determine the extraction temperatures that will preserve the stability of these phenolic substances [31]. Therefore, the amount of ISQ in the following experiments was maximized in the temperature range of 50–70 °C.

#### 3.1.4. Effect of Ultrasonication Time on ISQ Extraction

Another important factor affecting ISQ extraction is extraction time (Figure 2d). The amount of extracted ISQ increased with extraction time, peaking at 20 min. Although longer exposure can improve the extraction of ISQ by increasing its release into the solvent from the powder [6], it can also lead to its degradation [32]. Ultrasound extraction can degrade isolated ISQ; thus, prolonged ultrasonic exposure can oxidize ISQ. Hence, an extraction time between 10 and 40 min in the following experiments was chosen to save time and energy.

#### 3.1.5. Effect of Ultrasonic Power on ISQ Extraction

Ultrasonic energy showed a considerable impact on the extraction of ISQ (Figure 2e). At ultrasonic energies from 30 W to 60 W, the amount of extracted ISQ increased significantly with increasing ultrasonic energy, whereas it decreased with increasing ultrasonic energy above 60 W. An increase in ultrasound power can have a strong effect on the activity of cavitation bubbles. However, higher ultrasonic power may damage the ISQ structure due to the thermal effect, which contributes to a lower extraction rate [28]. It has been reported that the increase in cavity size due to higher ultrasonic power improves the extraction of polyphenols in the rind extract of *Nephelium lappaceum* fruit [30] and grape seeds [33]. Different levels of ultrasonic intensity have been used to extract polyphenols from tea leaves, resulting in a 16% increase in yield when the power level is increased from 25 to 125 W [33]. The efficiency of polyphenol extraction from *Acer truncatum* leaves increased by 9.5% when the power was increased from 150 to 240 W [34]. In addition, high ultrasound power can generate hydroxyl radicals (OH*) that react with and reduce phenolic compounds, particularly when the water content is high [35]. For this reason, care should be taken to prevent the degradation of polyphenols by high energy intake. Changes in the chemical composition of extracts related to the effects of high ultrasound power have also been observed in other studies. For this reason, in the following experiments, the ultrasound power was optimized between 60 W and 120 W.

### 3.2. Optimization of ISQ Extraction by RSM

#### 3.2.1. Response Surface Model Analysis

Based on the experimental results of the SFED, a Response Surface Method (RSM) experiment was designed for the optimization of the ISQ extraction method of *E. alata* (Table 2). Design-Expert 7.0.0 software was used to adjust the response value of the ISQ extraction by multiple linear regression. The equation is expressed as follows:

$$\begin{array}{ll} Y\left(\mu g/g\right) & =8.296-1.01\;X1-0.65\;X2+0.40\;X5+0.40\;X1X2\\ \; & +0.17\;X1X4-0.16\;X1X5-0.26\;X2-0.27\;X2X5\\ \; & +0.14\;X1^{2}-0.79\;X2^{2}+0.30\;X3^{2}+0.17\;X4^{2}+0.30\;X5^{2} \end{array}$$

where Y is the yield of ISQ (µg/g), and X1, X2, X3, X4, and X5 are the coded variables for ultrasonic time, ultrasonic temperature, solvent-to-solid ratio, ultrasonic power, and ethanol concentration, respectively.

The significance coefficient of the model and the results of the analysis of variance (ANOVA) are presented in Table 3. With a *p*-value < 0.0001, high significance was indicated for the regression model. The model terms X1, X2, X1X2, X2^2^, X3^2^, and X5^2^ were found to be highly significant (*p* < 0.0001). The model terms X1X4, X1X5, X2X4, X2X5, X1^2^, and X4^2^ were found to be significant (*p* < 0.05). The remaining terms were found to be non-significant (*p* > 0.10). The ANOVA indicated that the most significant conditions for the recovery of ISQ from *E. alata* were ultrasound temperature (with three significant interactions, X1X2, X2X4, and X2X5), ultrasound duration (with three significant interactions, X1X2, X1X4, and X1X5), and ethanol concentration (with two significant interaction conditions, X1X5, and X2X5). Correlation coefficient R1 was measured for the statistical relationship between actual and predicted points. High significance was indicated by the R with an absolute value of 99.24% and an adjusted R1 of 98.08%. The recovery of ISQ in the 46 runs ranged from 652.24 to 1033.96 µg/g (Table 2). The highest recovery of ISQ (1033.96 µg/g) was obtained under the following conditions (run 28): an ultrasonic time of 10 min, an ultrasonic temperature of 60 °C, a solvent-to-solid ratio of 60 mL/g, an ultrasonic power of 75 W and an ethanol concentration of 70%. The lowest ISQ recovery (652.24 µg/g) with the UAE method was obtained in run 17.

#### 3.2.2. Interactions between UAE Factors and the Response Surface

**Figure 3:**
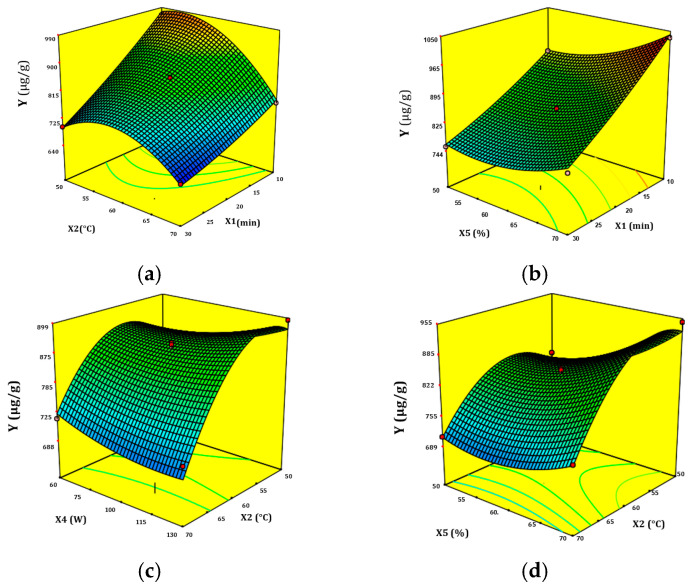
Response surface plots of the ISQ content (µg/g) of UAE as affected by the interaction between the pairwise factors: (**a**) ultrasonic time (X1) and ultrasonic temperature (X2), (**b**) EtOH concentration (X5) and ultrasonic time (X1), (**c**) ultrasonic power (X4) and ultrasonic temperature (X2), (**d**) EtOH concentration (X5) and ultrasonic temperature (X2).

The three-dimensional response surface plots illustrate the interaction between the pairwise factors (Figure 3a–d). In Figure 3a, the surface plot demonstrates the interaction between ultrasonic time (X1) and ultrasonic temperature (X2) and shows that the extraction yield of ISQ decreased with the simultaneous increase of the X1 and X2 factors. Therefore, by increasing the extraction time and continuously releasing the extracted products, the extraction solvent should be saturated [6]. A further increase in temperature can also accelerate the evaporation process. In this case, the extraction yield of ISQ can be decreased because the solvent surface area is reduced between the solid and the solvent. Figure 3b presents the surface plot for the interaction between EtOH concentration (X5) and ultrasonic time (X1). However, it is generally observed that when both parameters are decreased simultaneously, the maximum extraction yield increases. Due to the decrease in temperature, the evaporation rate of the solvent can be reduced, and the desired amount of solvent can be reduced. The addition of formic acid as a cosolvent can enhance the ISQ release into the solvent by improving the contact with the solid. As a result, the yield surface can be increased [36]. Figure 3c shows an interaction between ultrasonic power (X4) and ultrasonic temperature (X2). Furthermore, as the ultrasonic power increased, the extraction yields decreased at an ultrasonic temperature (X2) of 60 °C. The extraction yield of ISQ is generally reduced when both parameters (X4) and (X2) are increased simultaneously. The concentrations of solid fillers in the sample may increase as the solvent evaporates, which may result in a reduction in extraction efficiency due to the reduction in solvent/solid sample contact area. In addition, by using the higher ultrasonic power of UAE, the structure of bioactive compounds may be altered or destroyed [23]. Figure 3d shows how EtOH concentration (X5) and ultrasonic temperature (X2) affect the extraction performance of ISQ because of the response surface gradient. Thus, at 70 °C, as the EtOH concentration decreases, the response surface shows a decrease in extraction yield. However, at lower temperatures, the yield increases with increasing EtOH concentration. High temperatures can break down the structure of ISQ, resulting in a low extraction yield [37]. Generally, reducing the ultrasonic temperature while increasing the amount of solvent simultaneously improved the extraction yield surface.

### 3.3. Antioxidant and Enzyme Inhibitory Activities

Isoquercetin, a glycosidic form of quercetin, has been shown to possess antioxidant, neuroprotective, anti-inflammatory, and antidiabetic properties. Through activation of the Nrf2/ARE antioxidant signaling pathway, ISQ attenuates ethanol-induced hepatotoxicity, oxidative stress, and inflammatory responses [38]. By modulating nuclear factor B (NF-B), ISQ regulates nitric oxide synthase 2 (NO2) expression. It has been suggested that ISQ may prevent birth defects in diabetic pregnancies due to its high bioavailability and low toxicity [39]. In this study, the efficacy of ISQ-UAE (isoquercetin extracted by the optimized ultrasound-assisted extraction method) in scavenging radicals and their anti-hyperpigmentation, anti-aging, anti-diabetes, anti-inflammation, and anti-neurodegenerative disease qualities were evaluated and compared with ISQ-SE (isoquercetin extracted by Soxhlet) (Table 4).

Therefore, an in vitro evaluation of the antioxidant activities of ISQ was conducted. Three complementary bioassays were evaluated, including ABTS, FRAP, and DPPH (Table 4). The results indicate that ISQ-UAE exerts a high antioxidant potential in vitro (82.47, 88.93, and 77.15 mg TE/g for DPPH, FRAP, and ABTS tests, respectively) in contrast to ISQ-Soxhlet (64.81, 65.71, and 61.19 mg TE/g for DPPH, FRAP and ABTS tests, respectively).

Acetylcholinesterase (AchE) and butyrylcholinesterase (BchE) are two key enzymes involved in the management of certain pathologies, including Alzheimer’s disease (AD). Abnormal β-amyloid protein may be one of the key factors in AD progression, and cholinesterase promotes β-peptide aggregation and amyloid formation [40]. Therefore, for the treatment of AD, inhibiting the cholinesterase enzyme and preventing amyloid aggregation would be effective [41]. Scientific research has focused much attention on the search for safe natural enzyme inhibitors. In this study, the inhibition of cholinesterase (AchE and BchE) and Aβ1-42 peptide aggregation by ISQ from *E. alata* were tested. In our study, ISQ-UAE presented the highest potential enzyme inhibitory activities against AchE (1.56 mg GALAE/g), BchE (4.02 mg GALAE/g), and Aβ1-42 peptide aggregation (74.11%) compared with ISQ-SE (Table 4).

Diabetes mellitus (DM), a disorder of the endocrine system affecting carbohydrate metabolism, results in a rapid rise in blood glucose levels. The compound α-amylase is charged with transforming oligo- and disaccharides into monosaccharides while inhibiting the hydrolysis of carbohydrates to delay their absorption [42]. Thus, diabetes and obesity can be treated more effectively by optimizing postprandial blood glucose levels. The use of natural anti-amylase represents an interesting biotreatment option for hyper-glycemia. Therefore, in this study, we sought to determine the α-amylase inhibitory potency of ISQ from *E. alata* extracted by the optimized UAE method compared with ISQ-SE. Furthermore, ISQ-UAE also showed the highest anti-α-amylase activities compared to ISQ-SE (Table 4).

The extracellular matrix (ECM) is the infrastructure of the skin’s foundation and is composed of several structural components, such as collagen, elastin, and micro fibrils. As the skin matures, these constituents undergo a transformative process that is characterized by the appearance of dry, wrinkled, and lax skin [43]. Tyrosinase is a metalloprotein present in the membrane of the melanosome. This multi-functional protein is responsible for catalyzing the two first steps of melanin biosynthesis and is responsible for converting tyrosine into dopaquinone [44]. Collagen and elastin are important constituents of the ECM. These compounds contribute to skin elasticity and strength [44], and their degradation by collagenase and elastase is one of the main causes of intrinsic skin aging [45]. In this study, we found that the highest inhibition of tyrosinase, collagenase, and elastase was recorded with ISQ-UAE (95.04, 87.31, and 88.93%, respectively) compared to ISQ-SE (Table 4).

Hyaluronate (hyaluronic acid) plays important biological roles in humans. This acid is naturally synthesized by hyaluronan synthases and is degraded by a group of enzymes called hyaluronidases [46]. The ECM is hydrolyzed by hyaluronidase during tissue remodeling, and upregulation of hyaluronidase activity is observed in chronic inflammatory states [43,47]. It has been suggested that inhibitors of hyaluronidase have a beneficial effect in preventing and treating inflammatory diseases [47]. Thus, in this study, the hyaluronidase inhibitory activity of ISQ from *E. alata* was evaluated. ISQ-UAE presents promising anti-hyaluronidase activity compared with ISQ-SE (98.83 and 83.68%, respectively).

Our data showed that ISQ from *E. alata* presents promising results for inhibiting tyrosinase, elastase, collagenase, α-amylase, hyaluronidase, cholinesterase, and Aβ1-42 aggregation. ISQ-UAE had superior performance compared with that of ISQ-SE for all assays, which can be explained by the higher temperature (60 °C) and prolonged extraction time (6 h) of the Soxhlet method, which are likely to degrade ISQ, contributing substantially to a decrease in bioactivity [15,48,49].

### 3.4. Observation by Scanning Electron Microscopy

**Figure 4:**
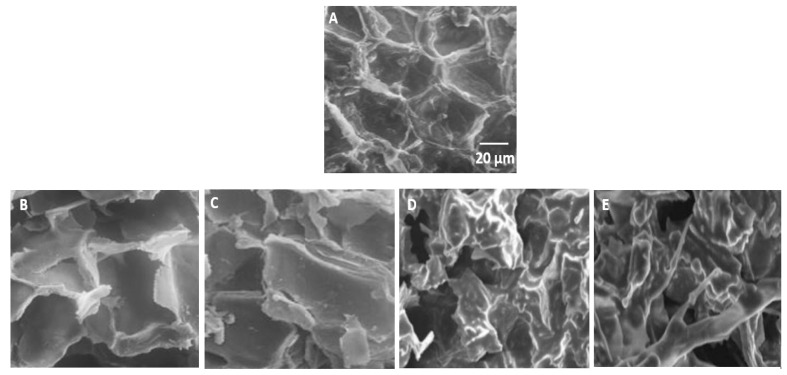
Scanning electron microscopy images of *E. alata* powder: control sample before extraction (**A**), Soxhlet (**B**,**C**), and UAE (**D**,**E**).

Scanning electron microscopy (SEM) was used to observe the structural changes that occurred during the different extraction processes to better understand the differences in the extraction mechanisms with the optimized UAE and Soxhlet methods. Figure 4 shows SEM images of the untreated sample and the sample after extraction by Soxhlet (Figure 4B,C) and UAE (Figure 4D,E). Extraction produced cellular changes in all samples compared to the control sample (Figure 4A), although the extent of damage differed among samples. The sample obtained after Soxhlet extraction showed slight pore disruption, whereas more marked changes were observed in the samples obtained after extraction by the optimized UAE method. The UAE treatment caused a disruption of the plant tissue and created several hollow openings, manifesting cavitation phenomena. In addition, the channels were destroyed, and more cracks and pores were formed by ultrasonic extraction. Therefore, the extraction of ISQ occurred at a higher rate with ultrasonic treatment. It was found that extraction from dried materials requires two steps: (i) the soaking of the plant materials in a solvent to promote swelling and hydration mechanisms and (ii) mass transport of the soluble components from the material to the solvent through diffusion and osmotic processes [50,51]. This suggests that the ultrasound treatment induced a subsequent change in the cellulose surface tension, with several pits occurring on the surface of the material (Figure 4). The modification of cellulose might cause the plant to crumble or disintegrate more rapidly. Moreover, pitting with UAE could promote diffusion and osmotic processes.

## 4. Conclusions

This study successfully applied the RSM method as a practical approach to optimizing UAE for the extraction of isoquercetin from *E. alata* to increase extraction efficiency and preserve bioactivities. Using a single-factor experimental design (SFED), the effects of five independent parameters (EtOH concentration, liquid-solid ratio, extraction time, extraction temperature, and ultrasonic power) on the extraction efficiency of isoquercetin from *E. alata* were evaluated. Following the results of the SFED study, optimization of the extraction conditions was performed by response surface methodology (RSM) using the Box–Behnken design (BBD). The highest recovery of ISQ (1033.96 µg/g) was obtained under the following conditions (cycle 28): ultrasonic time of 10 min, ultrasonic temperature of 60 °C, solvent/solid ratio of 60 mL/g, ultrasonic power of 75 W and ethanol concentration of 70%. In addition, ISQ from *E. alata* showed promising results for the inhibition of the key enzymes involved in hyperpigmentation, skin aging, diabetes, inflammation, and neurodegenerative diseases. Basically, for all the bioassays tested, the isoquercetin extracted by the optimized ultrasound-assisted extraction method (ISQ-UAE) was found to be more efficient than the isoquercetin extracted by Soxhlet extraction (ISQ-SE).

## Figures and Tables

**Table 1:** Independent variables at three coded levels of variation for the Box–Behnken design.

Variables	Label	Levels		
		−1	0	1
Time (min)	X1	10	20	30
Temperature (°C)	X2	50	60	70
Solvent/Solid ratio (mL/g)	X3	50	60	70
Ultrasound power (W)	X4	60	75	90
Ethanol concentration (%)	X5	50	60	70

**Table 2:** Box–Behnken design with independent coded variables and response variables.

Run	Independent Variables					ISQ (µg/g)
	X1 (min)	X2 (°C)	X3 (mL/g)	X4 (W)	X5 (%)	
**1**	10 (−1)	50 (−1)	60 (0)	75 (0)	60 (0)	955.66 ± 2.44
**2**	20 (0)	50 (−1)	60 (0)	75 (0)	70 (1)	939.67 ± 2.56
**3**	20 (0)	50 (−1)	50 (−1)	75 (0)	60 (0)	850.11 ± 1.88
**4**	30 (1)	60 (0)	60 (0)	60 (−1)	60 (0)	760.98 ± 1.09
**5**	20 (0)	60 (0)	60 (0)	75 (0)	60 (0)	850.90 ± 1.56
**6**	20 (0)	60 (0)	70 (1)	60 (−1)	60 (0)	878.55 ± 1.87
**7**	20 (0)	60 (0)	60 (0)	75 (0)	60 (0)	833.78 ± 2.09
**8**	10 (−1)	60 (0)	60 (0)	75 (0)	50 (−1)	921.89 ± 2.44
**9**	10 (−1)	60 (0)	60 (0)	90 (1)	60 (0)	943.09 ± 2.67
**10**	30 (1)	50 (−1)	60 (0)	75 (0)	60 (0)	701.33 ± 0.89
**11**	20 (0)	70 (1)	60 (0)	60 (−1)	60 (0)	721.56 ± 1.43
**12**	20 (0)	60 (0)	70 (1)	75 (0)	70 (1)	950.77 ± 2.43
**13**	30 (1)	60 (0)	60 (0)	75 (0)	70 (1)	795.67 ± 1.22
**14**	20 (0)	60 (0)	50 (−1)	90 (1)	60 (0)	873.78 ± 1.67
**15**	20 (0)	60 (0)	50 (−1)	60 (−1)	60 (0)	863.64 ± 1.98
**16**	20 (0)	70 (1)	71 (1)	75 (0)	60 (0)	728.38 ± 1.11
**17**	30 (1)	70 (1)	60 (0)	75 (0)	60 (0)	652.24 ± 1.09
**18**	20 (0)	60 (0)	60 (0)	90 (1)	50 (−1)	843.6 ± 1.90
**19**	20 (0)	60 (0)	60 (0)	75 (0)	60 (0)	841.6 ± 1.67
**20**	20 (0)	60 (0)	60 (0)	90 (1)	70 (1)	920.75 ± 2.78
**21**	10 (−1)	70 (1)	60 (0)	75 (0)	60 (0)	759.44 ± 1.22
**22**	10 (−1)	60 (0)	60 (0)	60 (−1)	60 (0)	993.88 ± 2.78
**23**	20 (0)	70 (1)	60 (0)	90 (1)	60 (0)	714.35 ± 1.11
**24**	30 (1)	60 (0)	60 (0)	75 (0)	50 (−1)	753.43 ± 0.89
**25**	20 (0)	60 (0)	70 (1)	90 (1)	60 (0)	917.74 ± 3.04
**26**	20 (0)	60 (0)	50 (−1)	75 (0)	70 (1)	947.8 ± 2.67
**27**	20 (0)	70 (1)	50 (−1)	75 (0)	60 (0)	724.37 ± 1.02
**28**	10 (−1)	60 (0)	60 (0)	75 (0)	70 (1)	1033.96 ± 3.28
**29**	30 (1)	60 (0)	70 (1)	75 (0)	60 (0)	796.51 ± 2.01
**30**	20 (0)	60 (0)	70 (1)	75 (0)	50 (−1)	862.64 ± 2.76
**31**	20 (0)	60 (0)	60 (0)	75 (0)	60 (0)	842.6 ± 2.64
**32**	20 (0)	50 (−1)	60 (0)	90 (1)	60 (0)	887.68 ± 2.01
**33**	10 (−1)	60 (0)	70 (1)	75 (0)	60 (0)	1002.9 ± 2.89
**34**	20 (0)	50 (−1)	60 (0)	60 (−1)	60 (0)	796.51 ± 1.11
**35**	20 (0)	60 (0)	60 (0)	60 (−1)	70 (1)	935.77 ± 2.56
**36**	10 (−1)	60 (0)	50 (−1)	75 (0)	60 (0)	999.9 ± 3.01
**37**	20 (0)	50 (−1)	60 (0)	75 (0)	50 (−1)	812.54 ± 2.12
**38**	30 (1)	60 (0)	60 (0)	90 (1)	60 (0)	778.48 ± 2.01
**39**	20 (0)	70 (1)	60 (0)	75 (0)	50 (−1)	712.35 ± 1.88
**40**	30 (1)	60 (0)	50 (−1)	75 (0)	60 (0)	785.49 ± 2.11
**41**	20 (0)	50 (−1)	70 (1)	75 (0)	60 (0)	853.62 ± 2.82
**42**	20 (0)	70 (1)	60 (0)	75 (0)	70 (1)	748.42 ± 1.12
**43**	20 (0)	60 (0)	60 (0)	75 (0)	60 (0)	849.61 ± 1.98
**44**	20 (0)	60 (0)	60 (0)	75 (0)	60 (0)	832.58 ± 2.67
**45**	20 (0)	60 (0)	50 (−1)	75 (0)	50 (−1)	863.64 ± 1.09
**46**	20 (0)	60 (0)	60 (0)	60 (−1)	50 (−1)	844.6 ± 0.99

**Table 3:** ANOVA of the central composite design.

Source	Sum of Squares	df	Mean Square	*F*-Value	*p*-Value	Significance
**Model**	37.7094	20	1.8870	86.8530	<0.0001	**
**X1**	16.0752	1	16.0752	740.5608	<0.0001	**
**X2**	6.8952	1	6.8952	317.6382	<0.0001	**
**X5**	2.8152	1	2.8152	129.4788	<0.0001	**
**X1X2**	0.6120	1	0.6120	28.2234	<0.0001	**
**X1X4**	0.0979	1	0.0979	4.5186	0.0465	*
**X1X5**	0.1122	1	0.1122	5.2734	0.0324	*
**X2X4**	0.2550	1	0.2550	11.5158	0.0026	*
**X2X5**	0.2346	1	0.2346	10.5978	0.0036	*
**X1^2^**	0.1122	1	0.1122	5.2122	0.0335	*
**X2^2^**	5.5896	1	5.5896	257.4072	<0.0001	**
**X3^2^**	0.8976	1	0.8976	41.1774	<0.0001	**
**X4^2^**	0.1734	1	0.1734	8.0580	0.0097	*
**X5^2^**	0.8772	1	0.8772	40.5246	<0.0001	**
**Residual**	0.5508	25	0.0224			
**Lack of Fit**	0.5202	20	0.0265	4.5798	0.0525	
**Pure Error**	0.0296	5	0.0058			
**Cor Total**	38.2602	45				
**Std. Dev.**	0.1530					
**R^2^**	0.9924					
**R^2^ Adj**	0.9808					

* significant; ** highly significant.

**Table 4:** Antioxidant and enzyme inhibitory activities of *E. alata* extracts.

Antioxidant and Enzyme Inhibitory Activities	ISQ-UAE	ISQ-SE
**DPPH (mg TE/ g)**	82.47 ± 1.55	64.81 ± 1.09
**FRAP (mg TE/g)**	88.93 ± 1.56	65.71 ± 1.56
**ABTS (mg TE/g)**	77.15 ± 2.08	61.19 ± 1.89
**AchE inhibition (mg GALAE/g)**	1.56 ± 0.12	1.15 ± 0.11
**BchE inhibition (mg GALAE/g)**	4.02 ± 0.23	3.45 ± 0.20
**Aβ_1–42_ inhibition (%)**	74.11 ± 0.49	66.87 ± 0.21
**Alpha-amylase inhibition (mmol ACE/g)**	0.15 ± 1.89	0.12 ± 0.01
**Tyrosinase inhibition (mg KAE/g)**	95.04 ± 3.56	62.93 ± 2.78
**Collagenase inhibition (%)**	87.31 ± 1.88	73.90 ± 1.77
**Elastase inhibition (%)**	88.93 ± 1.86	79.03 ± 1.89
**Hyaluronidase inhibition (%)**	98.83 ± 2.70	83.68 ± 3.67

Results are reported as mean ± SD (*n* = 3) and were significant (*p* < 0.05). ISQ extracted by the optimized ultrasound-assisted extraction (UAE) method (ISQ-UAE) and ISQ extracted by Soxhlet (ISQ-SE). ABTS, FRAP, and DPPH were expressed as Trolox equivalents per gram (mg TE/g). Acetylcholinesterase (AchE) and butyrylcholinesterase (BchE) inhibition capacities were expressed as milligrams of galantamine equivalent per gram (mg GALAE/g). The ability to inhibit α-amylase was reported in mmol acarbose equivalent per gram of sample (mmol ACE/g extract). The inhibition of tyrosinase is presented in kojic acid equivalent per gram of sample (mg KAE/g extract). The inhibitory capacities of collagenase, hyaluronidase, and elastase enzymes are reported in percent (%).

## Data Availability

Not applicable.
